# Simulation tools in neuro-oncological surgery: a scoping review of perioperative and training applications

**DOI:** 10.1007/s11060-025-04972-8

**Published:** 2025-03-19

**Authors:** Grazia Menna, Dora Riva, Salvatore Marino, Jocelyn Garber, Jasper Kees Wim Gerritsen, Pier Paolo Mattogno, Jacob Stewart Young, Alessandro Olivi, Francesco Doglietto, Mitchel Stuart Berger, Giuseppe Maria Della Pepa

**Affiliations:** 1 Neurosurgery Unit, Department of Neurosciences, Catholic University School of Medicine, Rome, Italy; 2 Neurosurgery Unit, Department of Neurosciences, Fondazione Policlinico Universitario Agostino Gemelli, Istituto di Ricovero e Cura a Carattere Scientifico (IRCCS), Rome, Italy; 3 University of California, Santa Barbara, USA; 4 Department of Neurosurgery, Erasmus Medical Center, Rotterdam, The Netherlands; 5 Depertment of Neurosurgery, University of California San Francisco, San Francisco, USA

## Abstract

**Background:**

Neuro-oncological surgery has lagged other neurosurgical subspecialties in integrating simulation technologies for training and surgical planning. This study provides a comprehensive scoping review of the current landscape of simulation tools in neuro-oncological surgery, mapping existing research, identifying technological advancements, and highlighting gaps in surgical training and perioperative planning.

**Methods:**

We formulated the research question: “What is the effect of perioperative simulation and neuro-oncological training on surgical skill acquisition, patient outcomes, and safety among neurosurgeons, compared to traditional or no training methods?” A comprehensive search was conducted on PubMed, Scopus and ClinicalTrials.gov, with the final search completed in May 2024. The quality of training studies was assessed using the Medical Education Research Study Quality Instrument (MERSQI), and the Cochrane ROBINS-I tool was used to evaluate bias in simulation studies.

**Results:**

The search yielded 5,518 records, with 51 studies meeting the inclusion criteria. These were categorized into six groups: (1) 3D Models in Presurgical Planning and Intraoperative Navigation: 5 articles; (2) Augmented Reality (AR) in Presurgical Planning and Intraoperative Navigation: 25 articles; (3) Mixed Reality (MR) in Presurgical Planning and Intraoperative Navigation: 6 articles; (4) Virtual Reality (VR) in Presurgical Planning and Intraoperative Navigation: 4 articles; (5) AR in Surgical Training: 5 articles; (6) VR in Surgical Training: 6 articles.

**Conclusion:**

While the number of studies on simulation in neuro-oncological surgery is increasing, their analytical depth remains limited. Simulation holds promise for advancing the field, but a significant journey lies ahead before achieving universal academic validation.

**Supplementary Information:**

The online version contains supplementary material available at 10.1007/s11060-025-04972-8.

## Introduction

Neurosurgery, like other medical and surgical fields, is increasingly benefiting from the integration of simulation technologies in both training and surgical planning. Modern tools such as virtual reality (VR), augmented reality (AR), and mixed reality (MR) [47] have started to complement traditional training methods that previously relied on animal models and human cadaver specimens (Table 1). These advanced technologies provide diverse, immersive experiences that not only significantly enhance the training of future neurosurgeons but also supports experienced surgeons in planning and executing complex procedures.

**Table 1:** Summary of types of simulation methods

	Definition	PROs	CONs	Examples
Cadaveric model	Animal or human either fresh, fresh frozen, injected head, brain hemisphere	Faithful anatomical representation	- Not reproducible or reusable - Costly and ethical issues	
Synthetic model	Bench mock-up of neurosurgical pathology using synthetic materials that mimic real tissues	Portable and reusable	Low-fidelity representation of pathology	
Augmented reality	Digital information (like images, text, or video) is overlaid on the real world, but these elements do not interact with or respond to the real-world environment. It enhances surgeon’s understanding of complex structures without altering their perception of the real world.	High-fidelity anatomical representation	Increase in operating time	UpSurgeOn Sina Trans-Visible navigator
Virtual reality	Varying degrees of immersion from interaction with video game-like console (non-immersive) to audio-visual headset (fully immersive)	Cost-effective	Hard to replicate haptic feedback	NeuroVR platform Immersive Touch
Mixed reality	In MR, digital and real-world objects coexist and can interact in real-time. 3D models of a patient’s anatomy could be manipulated as if they were real objects, seeing how these virtual elements interact with the real surgical field. It can be possible to adjusti the surgical approach based on real-time feedback from both the physical and digital elements.	Combination of AR and VR benefits	Simulated objects appear within and obey the boundaries of the real environment	HoloLens 2

Despite the clear benefits, published studies often lack clearly defined objectives and standardized metrics [56]. Although the number of studies published in neuro-oncology indicates growing interest and is comparable to other subspecialities, the range of simulation tools applied specifically to neuro-oncology is not as diverse or well-established [11, 44, 50]. Many of the published studies were preliminary in nature, with a focus on proof-of-concept rather than fully validated, large-scale applications [1, 5, 7, 17, 54].

This gap can be attributed to the inherent complexity of tumor pathology, the diversity of surgical approaches required, and the relatively recent development of targeted simulation technologies for this field. Thus, the aim of this study is to systematically review the current landscape of simulation tools utilized in neuro-oncological surgery. This review emphasizes advancements in simulation methodologies, particularly those involving augmented reality (AR), virtual reality (VR), and 3D technologies, which have demonstrated the most significant potential in enhancing neurosurgical education and practice.

## Methods

This scoping review follows the methodological framework proposed by Arksey and O’Malley, as further refined by Levac et al., and aligns with the PRISMA-ScR (PRISMA extension for Scoping Reviews) guidelines for reporting [36, 65]. 

Our approach includes systematically identifying, charting, and analyzing existing literature while mapping key concepts and gaps in the field of neuro-oncological simulation training and perioperative planning.

Our research question was: “What is the effect of perioperative simulation tools and neuro-oncological training on surgical skill acquisition, patient outcomes, and safety among neurosurgeons, compared to traditional or no training methods?” [47, 57].

We conducted a comprehensive search on:

- PubMed using the following terms: (“neuro-oncology” OR “glioma” OR “high-grade glioma”) AND (“training” OR “simulation” OR “virtual reality” OR “augmented reality”).
- Scopus using the following terms: (TITLE-ABS-KEY(“neuro-oncology” OR “glioma” OR “high-grade glioma” OR “brain tumor” OR “brain tumour”) AND TITLE-ABS-KEY(“simulation” OR “virtual reality” OR “augmented reality” OR “surgical training” OR “surgical simulation”).
- ClinicalTrials.gov using the following terms: (“neuro-oncology” OR “glioma” OR “high-grade glioma” OR “brain tumor” OR “brain tumor”) AND (“simulation” OR “surgical simulation” OR “virtual reality” OR “augmented reality” OR “mixed reality” OR “neurosurgical training” OR “operative simulation”).

The search was conducted up to May 2024. Two authors, D.R. and G.M., independently evaluated the papers, and any disputes were resolved by a third, senior author, G.M.D.P. Secondary searches involved reviewing the references of identified articles to uncover additional relevant studies.

Included studies were those published in English, centered on neurosurgical oncology, and described simulation-based interventions such as pre-surgical planning of tumor resections, intraoperative image-guided neuronavigation, or skills acquisition in a training environment. We considered digital simulations (AR, VR, mobile platforms) and physical simulations (3D printing models). Exclusions were made for publications not focused on neurosurgical simulations, those discussing other surgical fields without relevance to neuro-oncology, or those that did not report specific outcomes. There were no restrictions regarding the publication date.

The integrity of the studies on training were evaluated using the Medical Education Research Study Quality Instrument (MERSQI) [56]. Additionally, the Cochrane ROBINS-I tool, designed for assessing the risk of bias in non-randomized studies of interventions, was applied to review preoperative and intraoperative simulation studies [62]. 

## Results

A comprehensive literature search across multiple databases yielded 5,518 records, distributed as follows: PubMed: 1,978 records, Scopus: 3,512 records.

ClinicalTrials.gov: 3 records, Citation Cross-Searching: 25 records.

**Fig. 1:**
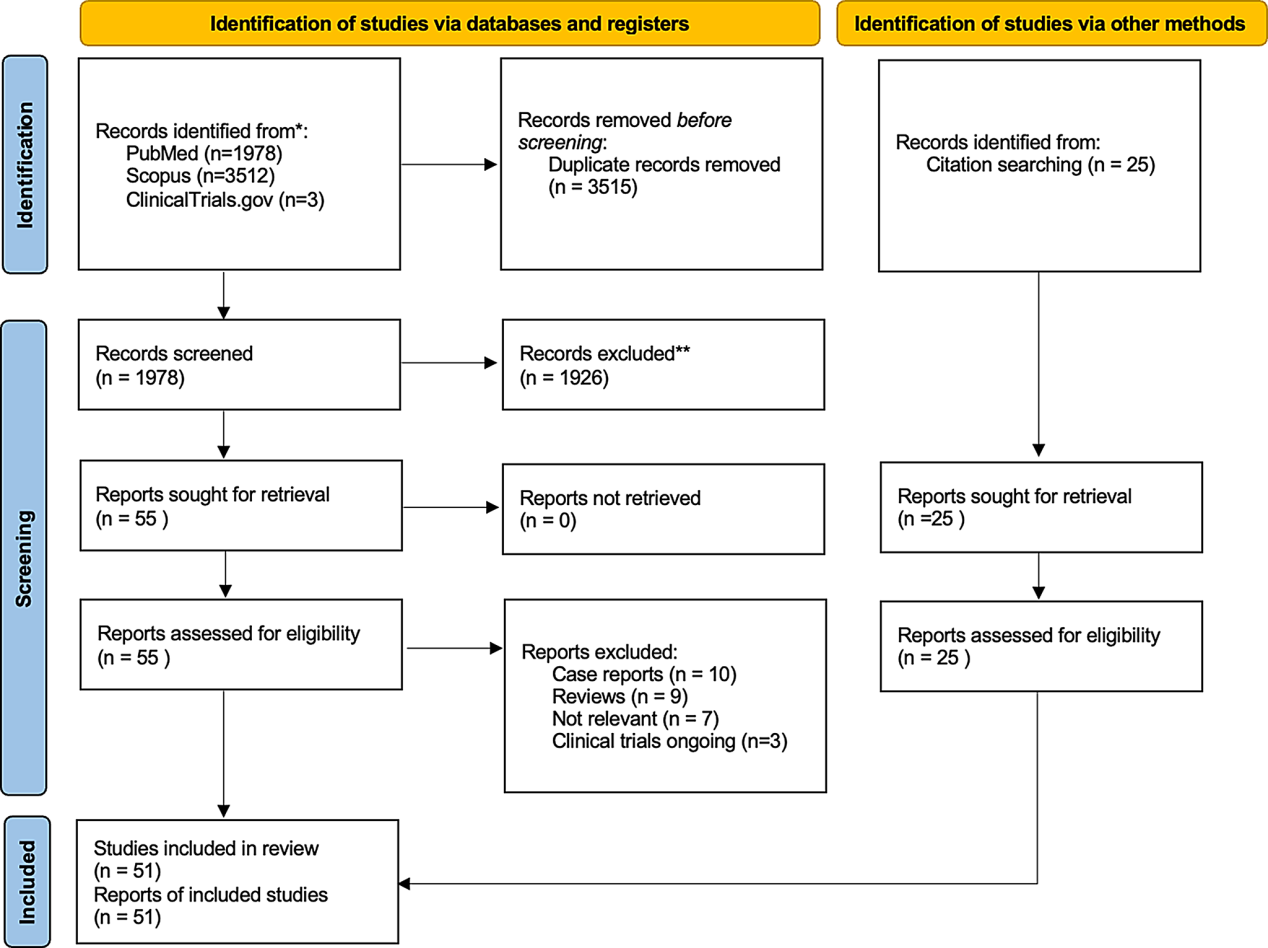
PRISMA 2020 flow diagram

After removing 3,515 duplicate records, 2,003 unique records remained for screening. Of the 2,003 screened records, 1,926 were excluded based on titles and abstracts. This left 55 full-text articles for eligibility assessment. Among the 55 full-text articles assessed: 10 were excluded as case reports, 9 were review articles, 7 were deemed irrelevant to the study objectives: 3 were clinical trials from ClinicalTrials.gov. These trials focus on the effects of intelligent-induced pausing on learning surgical skills (NCT06235788), the efficacy of verbal intelligent tutor instruction in neurosurgical simulation (NCT06273579), and neurosurgical skill enhancement through Transcranial Simulation (LETS-LEARN) (NCT02987816). and were not included in the final dataset as results were not yet posted. (Fig. 1, **PRISMA**).

As results, 51 studies met our inclusion criteria and were included in the analysis, categorized into the following groups:

1. 3D Models in Presurgical Planning and Intraoperative Navigation: 5 articles [20, 22, 33, 46, 64].
2. Augmented Reality (AR) in Presurgical Planning and Intraoperative Navigation: 25 articles [6, 8–10, 13, 15, 16, 18, 19, 25, 28, 29, 40, 50, 51, 59–61, 63, 66, 67, 69].
3. Mixed Reality (MR) in Presurgical Planning and Intraoperative Navigation: 6 articles [27, 30, 32, 39, 52, 70].
4. Virtual Reality (VR) in Presurgical Planning and Intraoperative Navigation: 4 articles [12, 21, 37, 53].
5. AR in Surgical Training: 5 articles [23, 26, 34, 35, 55].
6. VR in Surgical Training: 6 articles [2–4, 14, 24, 68].

### 3D models in presurgical planning and intraoperative navigation

The application of 3D models created using advanced printing technologies was notably underrepresented, with only 5 out of the 51 articles (9.8%) specifically addressing this area (Table 2) [20, 22, 33, 46, 64]. These studies primarily focused on the advantages of 3D models in presurgical planning. In these studies, 3D-printed models were utilized to accurately replicate patient-specific surgical scenarios. By recreating the tumor and surrounding brain structures based on preoperative imaging, these models provided a detailed visualization of the relationship between the neoplasm and surrounding anatomy. This allowed surgeons to optimize surgical strategies by preemptively identifying the safest approaches and minimizing risks to vital structures during the actual procedure.

**Table 2:** Paper regarding the use of 3D models in presurgical planning

Authors	Year	Location	Study design	Simulator discussed	Measurement of outcomes	*P*-value	Neurosurgical area of simulation
Guo et al. 2020 [22]	2020	CHINA	Case control (35 participants)	3-dimensional (3D)-printed models of skull base meningioma (Stratasys printer)	3D-printed skull base meningioma models for surgical planning and patient education. Comparison between the two groups	*p* = 0.321 (extent of resection) *p* = 0.138 (complications) *p* = 0.258 (function improvement) *p* = 0.208 (operating time) *p* = 0.280 (blood loss) *p* = 0.157 (length of stays)	Cranial neurosurgery: neurooncology
Okonogi et al. 2017 [46]	2017	JAPAN	Case series (51 participants)	a rapid prototyping (RP) model derived from the imaging data using a 3D printer	A 3D synthetic image from CT angiography and MRI data was compared to a 3D-printed rapid prototyping model. The model accurately reproduced the anatomy.	*p* < 0.17 (reproducibility of the lesion)	Cranial neurosurgery: neurooncology
Lan et al. 2019 [33]	2019	CHINA	Case series (7 participants)	3D-printed intracranial lesion models (Stratasys printer)	49 simulated surgeries using 3D-printed craniocerebral models. Actual surgeries were carried out after.		Cranial neurosurgery: neurooncology, neurovascular
Gomez-Feria et al. 2020 [20]	2020	SPAIN	Case series (2 participants)	3D-printed head models	3D-printed head models employed in 2 complicated DLGG surgeries. A questionnaire was administered		Cranial neurosurgery: neurooncology
Thawani et al. 2017 [64]	2017	USA	Survey-based design (3 participants)	3-dimensional (3-D) printing model of individualized tumors	A survey to assess the utility of the technique was administered to 4 faculty members and 5 residents		Cranial neurosurgery: neurooncology

Geographically, most of these studies were conducted in Asia [22, 33, 46], employing diverse study designs including one case-control study [22], one survey-based study [64], and three case series [20, 33, 44]. Both objective and subjective outcome measures were used to assess the efficacy of 3D models. Objective metrics included the extent of tumor resection, complication rates, and intraoperative blood loss, while subjective evaluations were gathered through surgeon questionnaires [20, 64].

Overall, although most studies demonstrated a low risk of bias in several domains, some highlighted significant gaps in reporting, particularly regarding confounding factors and incomplete data. (Supplementary Fig. 1)

### Augmented reality (AR) in presurgical planning and intraoperative navigation

Augmented Reality (AR) emerged as the most frequently studied technology, with 25 out of 51 articles (49.0%) addressing its use (Table 3). Among the 25 studies: 72% (18/25) focused on tumor localization and intraoperative guidance. 28% (7/25) explored AR-based neurosurgical planning. Several groups have integrated augmented reality navigation (ARN) into neurosurgical oncology to help with tumor localization. Hou et al. [25] used an iPhone-based app to locate the sagittal and axial projection of lesions in 35 patients, while Shu et al. [61] used an AR smartphone app to locate supratentorial lesions in 14 patients. The study by Hou et al. demonstrated that ARN could achieve high accuracy, although not suitable for deeper sub-occipital lesions or very small intraparenchymal lesions (< 1.5 cm).

**Table 3:** Papers regarding the use of AR in presurgical planning and intraoperative navigation

Authors	Year	Location	Study design	Simulator discussed	Measurement of outcomes	*P*-value	Neurosurgical area of simulation
Hou et al. 2016 [25]	2016	CHINA	Case series (35 participants)	A Low-Cost iPhone-Assisted Augmented Reality system	Check-point deviations between the iPhone-based method and CT (*p* = 0.75) or between the iPhone-based method and a frameless neurosurgical system (*p* = 0.30)	*p* = 0.75 (check-point deviations iPhone VS CT) *p* = 0.30 (check-point deviations iPhone VS frameless system)	Cranial neurosurgery: neurooncology, vascular neurosurgery
Shu et al. 2021 [61]	2021	CHINA	Case series (14 participants)	A smartphone augmented reality (AR) application (app)	Standard neuronavigation was applied to evaluate the accuracy of the smartphone. Max-margin distance (MMD) and area overlap ratio (AOR) were measured		Cranial neurosurgery: neurooncology
Satoh et al. 2021 [60]	2021	JAPAN	Case series (20 participants)	Trans-visible navigator, an Augmented Reality system	Screen capture videos of the tablet PC during surgery were recorded. Surgeons reviewed the videos after the surgery		Cranial neurosurgery: neurooncology
Eftekhar et al. 2016 [13]	2016	AUSTRALIA	Case series (11 participants)	Sina smartphone app (Augmented reality system)	Deviations between Sina localization and standard neuronavigation		Cranial neurosurgery: neurooncology
Watanabe et al. 2016 [67]	2016	JAPAN	Case series (6 participants)	Trans-visible navigator, an Augmented Reality system	an augmented reality-based navigation system with whole-operation-room tracking. Spatial resolution of about 1 mm.		Cranial neurosurgery: neurooncology
Sun et al. 2016 [63]	2016	CHINA	Case control (134 participants)	Virtual and augmented reality protocols based on functional neuronavigation and intraoperative MRI	Comparison between the two groups regarding the complete resection rate and average extent of resection (*p* = 0.01), and the rate of preservation of neural functions (*p* = 0.01)	*p* = 0.01 (resection rate, extent of resection, and rate of preservation of neural functions)	Cranial neurosurgery: neurooncology
Carl et al. 2019 [9]	2019	GERMANY	Case series (288 participants)	microscope-based AR support	iCT-based registration significantly (*P* < 0.001) increased AR accuracy	*p* < 0.001 (AR accuracy)	Cranial neurosurgery: neurooncology
Chidambaram et al. 2023 [10]	2023	USA	Case series (3 participants)	Intraoperative augmented reality fiber tractography	augmented reality and HDFT to facilitate direct visualization of the fiber tracts that are being stimulated		Cranial neurosurgery: neurooncology
Pennacchietti et al. 2021 [50]	2021	GERMANY	Case series (11 participants)	augmented reality neuronavigation in endoscopic assisted skull base pathologies	Clinical conditions, MRI appearance, intraoperative conditions, postoperative MRI, possible complications, and outcomes were analyzed		Cranial neurosurgery
Fick et al. 2021 [15]	2021	NETHERLANDS	Case series (3 participants)	Holographic neuronavigation using an augmented reality head-mounted display (AR-HMD)	The fiducial registration error (FRE) was measured as the outcome measure for registration accuracy		Cranial neurosurgery: neurooncology
Pojski et al. 2022 [51]	2022	GERMANY	Case series (39 participants)	Microscope-Based Augmented Reality	Combination of microscope-based AR and automatic patient registration using iCT		Cranial neurosurgery: neurooncology
Gerard et al. 2018 [19]	2018	CANADA	Experimental pilot study (8 participants)	A combination of intraoperative ultrasound and AR	Feasibility and potential benefits of combining iUS-based registration with AR to enhance intraoperative planning and visualization in neurosurgical procedures.	Average improvement was 68%.	Cranial neurosurgery: neurooncology
Besharati Tabrizi et al. 2015 [6]	2015	GERMANY	Case series (5 participants)	augmented reality system for image-guided neurosurgery	Comparison between a neuronavigation system (StealthStation, Medtronic Inc.) and AR	*p* = 0.3 (accuracy in tumor localization)	Cranial neurosurgery: neurooncology
Ivan et al. 2021 [29]	2021	USA	Case series (11 participants)	HoloLens, an Augmented Reality system	Tumor border tracing using HoloLens VS using StealthStation (correspondence degree)	*p* = 0.344 (correspondence degree)	Cranial neurosurgery: neurooncology
Luzzi et al. 2024 [42]	2024	ITALY	Case control (65 participants)	augmented reality fiber tractography	NANO and MRC scores as outcome measures of the safety of the technique, whereas EOR and survival curves were related to its cytoreductive efficacy	*p* < 0.05 (NANO, MRC, EOR, and survival curves)	Cranial neurosurgery: neurooncology
Luzzi et al. 2021 [41]	2021	ITALY	Case control (54 participants)	AR HDFT F	NANO score, EOR, and Kaplan-Meier curves as outcome measures	*p* < 0.05 (NANO, EOR)	Cranial neurosurgery: neurooncology
Fick et al. 2021 [16]	2021	NETHERLANDS	Case series (50 participants)	an automatic segmentation algorithm that generates 3D models of skin, brain, ventricles, and contrast-enhancing tumor from a single T1-weighted MR sequence	Comparison of the automatic segmentation algorithm for brain tumor with a manually segmented ground truth set		Cranial neurosurgery: neurooncology
Mascitelli et al. 2017 [43]	2017	USA	Case series (79 participants)	Navigation-Linked Heads-Up Display	Depth accuracy of the lesions was measured	*p* = 0,29 (deep VS superficial lesions accuracy)	Cranial neurosurgery: neurooncology, vascular neurosurgery
van Doormaal et al. 2019 [66]	2019	NETHERLANDS	Case series (3 participants)	neuronavigation system on commercially available smart glasses (HoloLens)	The fiducial registration error (FRE) was measured for both holographic neuronavigation (HN) and conventional neuronavigation (CN)		Cranial neurosurgery: neurooncology
Yavas et al. 2021 [69]	2021	TURKEY	Case series (8 participants)	a 3D-printed marker–assisted augmented reality (AR) neuronavigation using mobile devices	AR compared with optical tracking neuronavigation systems (OTNSs)	*p* < 0.05 (registration and intraoperative preparation time)	Cranial neurosurgery: neurooncology
Satoh et al. 2019 [59]	2019	JAPAN	Case series (5 participants)	Trans-visible navigator (AR)	Application of AR to Stereotactic Biopsy. Selection of optimal puncture trajectory was the outcome		Cranial neurosurgery
Louis et al. 2021 [40]	2021	USA	Case series (49 participants)	Virtual and Synchronized Augmented Reality Platform for Preoperative Planning and Intraoperative Navigation (SyncAR)	Custom 360◦ models from preoperative imaging for preoperative planning. Overlay opacity was measured		Cranial neurosurgery: neurooncology, vascular neurosurgery
Inoue et al. 2013 [28]	2013	JAPAN	Case series (3 participants)	An augmented reality (AR) neuronavigation system with Web cameras	Validation of the utility of this AR navigation system by superimposing tumors and vessels that had been segmented in advance onto a Web camera image		Cranial neurosurgery: neurooncology
Carl et al. 2019 [8]	2019	GERMANY	Case series (10 participants)	Microscope-based augmented reality (AR)	The overall AR registration error was 0.72 ± 0.24 mm (mean ± standard deviation)		Spinal neurosurgery
Finger et al. 2017 [18]	2017	GERMANY	Case series (29 participants)	augmented reality-enhanced navigated neuroendoscopy	Patients were evaluated for postoperative imaging, reoperations, and possible complications		Cranial neurosurgery

In addition to these low-cost systems, various AR simulators, such as the Trans-visible navigator [60], Sina system [13], and HoloLens [29] were also utilized. Notably, Fick et al. evaluated the effectiveness of an AR head-mounted displays (HMDs) [15]. The system offered two primary advantages over traditional neuronavigation systems. First, it rendered anatomical structures in stereoscopic 3D, eliminating the need for surgeons to mentally convert 2D images into the 3D surgical field, thus reducing interpretation errors and allowing them to focus on other critical tasks. Second, by directly superimposing images onto the surgical field, it minimized attention shifts, thereby increasing surgical efficiency. These technologies were found to be accurate, intuitive, and user-friendly, with no significant increase in operating time, as shown in Yavas et al.‘s study [69].

Nonetheless, a notable limitation across most studies was that the AR simulators were tested on a small subset of neurosurgical patients, which restricts the generalizability of their reported utility and effectiveness [6, 8, 10, 15, 19, 28, 29, 50, 60, 66, 67, 69].

Overall, while many studies maintained a low risk of bias in several domains, concerns about confounding factors and variability in bias were still present. (Supplementary Fig. 2)

### Mixed reality (MR) in presurgical and intraoperative settings

Six out of the 51 (11.8%) articles focused on Mixed Reality (MR) in presurgical or intraoperative settings, as summarized in Table 4. Among these 6 studies: 33% (2/6) discussed MR for surgical planning [27, 50], 67% (4/6) addressed MR in intraoperative navigation [27, 30, 32, 39, 52, 70]. 

**Table 4:** Papers regarding the use of MR in presurgical planning and intraoperative navigation

Authors	Year	Location	Study design	Simulator discussed	Measurement of outcomes	*P*-value	Neurosurgical area of simulation
Incekara et al. 2018 [27]	2018	NETHERLANDS	Case series (25 participants)	Magnetic resonance imaging-based 3-dimensional holograms of the patient’s head and tumor using the Hololens (MR)	Accuracy of the Hololens localization was assessed using neuronavigation as the gold standard	*p* < 0.0001 (deviation between HoloLens and neuronavigation)	Cranial neurosurgery: neurooncology
Qi et al. 2021 [52]	2021	CHINA	Case series (37 participants)	mixed-reality neuronavigation system (Hololens) with a wearable head-mounted device (HMD)	The contour of the holograms was compared with standard neuronavigation (FRE *p* < 0.001)	*P* < 0.001 (FRE)	Cranial neurosurgery: neurooncology
Koike et al. 2021 [32]	2021	JAPAN	Case series (16 participants)	Mixed reality computer graphic system	Alignment accuracy of the MR system was measured		Cranial neurosurgery: neurooncology
Jain et al. 2023 [30]	2023	SINGAPORE	Case series (3 participants)	HoloLens 2 mixed reality system	Comparison between HoloLens 2 and neuronavigation systems. Good correlation with the localization of the tumor		Cranial neurosurgery: neurooncology
Zhou et al. 2022 [70]	2022	CHINA	Case series (16 participants)	mixed reality-based multimodality-fused surgical navigation system	Comparison between phantom experiments and clinical applications (spatial registration error, total time required for the registration process, and Intraoperative superimposition error)		Cranial neurosurgery: neurooncology
Liu et al. 2022 [39]	2022	CHINA	Case control (53 participants)	mixed-reality (MR) holographic imaging technology	The rate of complete resection of tumor lesions (*p* = 0.001) and the evaluation accuracy of complete resection (*p* = 0.000) were compared between the two groups	*p* = 0.001 (complete resection of the tumor) *p* = 0,000 (accuracy of complete resection)	Spinal neurosurgery

Most studies were case series (83%), with HoloLens being the primary MR system evaluated. Similarly to AR, MR has shown significant benefits in the pre/intraoperative planning [27, 52]. However, unlike AR, which overlays digital content onto the real world without interacting with it, MR creates a new environment where real and virtual elements are fully integrated and can interact.

The primary outcomes assessed were: accuracy of MR systems vs. standard neuronavigation (50% of studies) and user experience and interface efficiency (33% of studies). However, several challenges are associated with the current MR systems, such as the reliance on 2D Image Display that lacks depth and spatial context, limited visualization fields, re-registration issues (where imaging data does not perfectly align with the patient’s anatomy during surgery), and difficulties in accurately visualizing or tracking surgical instruments within the operative field.

Overall, the risk of bias assessment revealed concerns regarding confounding factors and participant selection, indicating a need for more comprehensive reporting and standardized methodologies (Supplementary Fig. 3). (Supplementary Fig. 3)

### VR in presurgical planning and intraoperative navigation

Virtual Reality (VR) was explored in 4 out of 51 studies (7.8%) (Table 5), with 2 studies focusing on preoperative planning (50%) [21, 53] and 2 on intraoperative applications (50%) [12, 37].

**Table 5:** Papers regarding the use of VR in presurgical planning and intraoperative navigation

Authors	Year	Location	Study design	Simulator discussed	Measurement of outcomes	*P*-value	Neurosurgical area of simulation
Gosal et al. 2021 [21]	2021	INDIA	Case series (6 participants)	3D VR technique using RadiAnt software	Identification of the operative corridor was the objective		Cranial neurosurgery: neurooncology
Qiu et al. 2010 [53]	2010	CHINA	Case series (45 participants)	integrated 3-D stereoscopic visualization of structural MRI and DTI tractography	Postoperative motor function, the extent of tumor resection, and the modality and integrity of the pyramidal tract (PT) were evaluated		Cranial neurosurgery: neurooncology
Delion et al. 2020 [12]	2020	FRANCE	Case series (30 participants)	a Samsung Gear VHR (Samsung, Seoul, South Korea) combined with a Samsung S7 smartphone (android platform) and headphones	Language mapping was performed with a naming task, DO 80, presented on a digital tablet and then in two-dimensional and three-dimensional formats through a VRH. A questionnaire was then administered		Cranial neurosurgery: neurooncology
Liu et al. 2022 [37]	2022	CHINA	Case control (77 participants)	Combination of 3D Slicer preoperative planning and intraoperative mobile phone VR technology	Patients surgery indicators and Karnofsky Performance Scale (KPS) scores were compared between the two groups	*p* < 0,05 (surgical indicators) *p* < 0.001 (KPS score)	Cranial neurosurgery: neurooncology

While VR showed high effectiveness, concerns about confounding factors and participant selection bias were noted (Supplementary Fig. 4).

The primary objective of these studies was to better identify operative corridors. By leveraging 3D VR technology, large datasets from modern CT and MR scanners can be visualized within a fully interactive 3D virtual space. This technique is supported by several DICOM viewer software programs, including OsiriX, Horos, Slicer, and RadiAnt, which allow for detailed exploration of various aspects of standard CT/MRI datasets. Additionally, these platforms allow surgeons to import 3D visualizations into mobile VR applications, enabling the use of VR headsets during surgery for real-time integration of images with the patient’s anatomy [12, 21, 37, 53].

In the study by Qiu et al. [53], the use of magnetic resonance diffusion tensor imaging (DTI) enabled the visualization of pyramidal tract (PT) within the VR environment.

Similarly, Delion et al. [12] introduced an innovative use of VR in patients undergoing awake craniotomies. In their study, 30 patients with brain tumors near language centers participated in language mapping tasks (DO 80) via digital tablets and VR headsets. At the end of the surgery, patients were offered an additional interactive VR experiences, which included virtual movements and avatar interactions guided by a neuropsychologist.

Overall, while many studies demonstrated a low risk of bias, challenges related to confounding factors and participant selection were identified, suggesting a need for more rigorous and standardized reporting. (Supplementary Fig. 4)

### AR in surgical training

Simulation in neurosurgical training was primarily AR-based, with 5 out of 51 studies (9.8%) focusing on its role (Table 6) [23, 27, 34, 35, 55]. Among these 5 studies, 80% (4/5) involved neurosurgeons-in-training.

**Table 6:** Papers regarding the use of AR in surgical training

Authors	Year	Location	Study design	Simulator discussed	Measurement of outcomes	*P*-value	Neurosurgical area of simulation	Mersqi
Ille et al. 2020 [26]	2020	GERMANY	Cohort study: survey based design (15 participants)	Virtual dissection of the prepared DTI FTs using a spatial computer and AR goggles	The study aims to test and evaluate a new method for fiber dissection using augmented reality (AR). Questionnaire-based study		Cranial neurosurgery: neurooncology	8
Raffa et al. 2023 [55]	2023	ITALY	Survey based design (441 participants)	Survey Questionnaire	Perceived Value: Subjective evaluations of the usefulness of different technologies, including 3D visualization software and simulation tools based on augmented/mixed/virtual reality.		General neurosurgery	8
Léger et al. 2017 [34]	2017	CANADA	Cohort study: survey based design (12 participants)	Mobile AR and desktop AR	The impact of two different types of AR IGS set-ups (mobile AR and desktop AR) and traditional navigation on attention shifts. Significant difference (*p* < 0.0001) in attention shifts between traditional navigation, but no significant difference between the different AR set-ups.	*p* < 0.0001 (attention shift)	Cranial neurosurgery: neurooncology	10
Léger et al. 2020 [35]	2020	CANADA	Cohort study: experimental based (17 participants)	MARIN (mobile augmented reality interactive neuronavigation system)	Comparison in time (*p* < 0.0004) and accuracy (*p* < 0.04) between MARIN and neuronavigation	*p* = 0.0004 (time) *p* = 0.04 (accuracy)	Cranial neurosurgery: neurooncology	12
Gurses et al. 2024 [23]	2024	TURKEY	Cohort study: experimental (240 participants)	AR and VR-based educational models	Knowledge levels on white matter were assessed through a mini test administered to residents and medical students	*p* = 0.001 (correct answer rate)	Cranial neurosurgery: neurooncology	13

These studies, mainly cohort studies, revolved around neurosurgeons with varying levels of expertise and their experience with AR simulators, such as the MARIN system, as a training tool. The primary focus was to identify white matter tracts to improve fiber dissection skills. While these studies demonstrate a growing interest in white matter simulation, the limited number of articles indicates a gap in this area that needs to be addressed.

A notable advancement in AR training was presented by Gurses et al. [23], who developed AR and VR-based educational models for neuroanatomy learning. Leveraging advanced photogrammetry, they created detailed 3D models for educational use among neurosurgery residents and second-year medical students in Turkey, where cadaveric training had been disrupted due to an earthquake. Participants engaged with and evaluated the AR and VR models, completing a 20-item user experience survey. Additionally, a 10-question mini-test was conducted before and after the training sessions to assess knowledge acquisition and the achievement of learning objectives. The results indicated a significant improvement in both residents’ and students’ performance following the simulation training. However, 80% (4/5) of studies relied on subjective assessments, highlighting a need for objective proficiency metrics in future research (Supplementary Fig. 5) with the exception of the study by Léger et al. [34], where both the system’s time efficiency and accuracy were quantitatively measured and compared to those of a standard neuronavigation system. This indicates a need for more robust, objective assessments in future research to validate the effectiveness of AR-based training tools. (Supplementary Fig. 5)

### Virtual reality in surgical training

Virtual reality (VR) in surgical training was explored in 6 out of 51 studies (11.8%), as summarized in Table 7 [2–4, 14, 24, 68]. All these studies utilized the NeuroTouch system, also known as NeuroVR, which is specifically designed for craniotomy. This system provides both haptic and graphic feedback, making it a valuable tool for neurosurgery training programs in universities and hospitals.

**Table 7:** Papers regarding the use of VR in surgical training

Authors	Year	Location	Study design	Simulator discussed	Measurement of outcomes	*P*-value	neurosurgical area of simulation	Mersqi
AlZhrani et al. 2015 [3]	2015	CANADA	Cohort study: Assessment based (33 participants)	NeuroTouch Virtual Reality	A study to assess neurosurgical performance during the resection of simulated virtual reality tumors		Cranial neurosurgery: neurooncology	13,5
Winkler-Schwartz et al. 2016 [68]	2016	CANADA	Cohort study: Assessment based (16 participants)	NeuroTouch Virtual Reality	A study to assess bimanual psychomotor performance using previously validated metrics. Performances compared with those of a control group from a previous study	*p* = 0,07 (bimanual psychomotor performance)	Cranial neurosurgery: neurooncology	12,5
Alotaibi et al. 2015 [2]	2015	CANADA	Cohort study: Assessment based (18 participants)	NeuroTouch Virtual Reality	Assessing bimanual performance. Both objective metrics and a Likert questionnaire were used	*P* < 0,5 (bimanual performance)	Cranial neurosurgery: neurooncology	12,5
Bajunaid et al. 2017 [4]	2017	CANADA	Cohort study: Assessment based (24 participants)	NeuroTouch Virtual Reality	The impact of a simulated stressful virtual reality tumor resection scenario through The State-Trait Anxiety Inventory (STAI) questionnaire		Cranial neurosurgery: neurooncology	12,5
Holloway et al. 2015 [24]	2015	USA	Cohort study: Experimental based (83 participants)	NeuroTouch Virtual Reality	Comparison between experienced and inexperienced neurosurgeons in a virtual reality brain surgery simulator environment	*p* = 0.0286 (volume of tumor removed) *p* = 1 (volume of healthy brain removed) *p* = 0.0286 (surgical effectiveness) *p* = 0.286 (surgical efficiency)	Cranial neurosurgery: neurooncology	13,5
Fazlollahi et al. 2023 [14]	2023	CANADA	Randomized controlled trial (60 participants)	NeuroVR (CAE Healthcare) virtual reality simulator	Surgical performance metrics of medical students exposed to an AI-enhanced training curriculum were compared with a control group of participants who received no feedback and with expert benchmarks.	*p* < 0.01 (safety metrics)	Cranial neurosurgery: neurooncology	13,5

The primary focus of these cohort studies was to demonstrate how VR simulation could enhance the training and evaluation of technical skills that rely heavily on tactile and visual feedback. These studies involved neurosurgeons with varying levels of experience and assessed key surgical performance metrics, such as bimanual psychomotor proficiency.

VR has proven to be a highly effective tool in simulation training by significantly lowering cognitive load and reducing operative stress duration. The VR environment enabled precise quantification of surgeon performance, facilitates assessment of surgical proficiency, and supports the tracking of trainees’ progress over time. (Supplementary Fig. 6).

## Discussion

Although there is growing interest, the range of simulation tools applied in neuro-oncology is neither as diverse nor as advanced as in other subspecialties [23]. While our review included 51 studies—a relatively substantial number compared to other systematic reviews in neurosurgical simulation [11, 44, 48]—it is essential to highlight that the depth of research specific to neuro-oncological simulation remains limited.

Our systematic review categorized the studies into six groups, revealing distinct roles for various simulation technologies in neuro-oncological surgery. 3D-printed models were particularly effective in presurgical planning. However, a major drawback of 3D printing is the significant time and resources required to produce these physical models. In contrast, AR was primarily employed for intraoperative navigation, where its ability to overlay real-time imaging onto the surgical field was the most frequently discussed application. VR and MR offered deeper integration by allowing real-time interaction between virtual elements and the physical environment, improving spatial awareness. While these tools contribute to enhancing surgical skills, patient safety, and outcomes, their effectiveness varied significantly based on the context—whether in presurgical planning or intraoperative navigation. [31, 45, 49, 58]

Simulation technologies aimed at training were less explored compared to those for presurgical planning and intraoperative guidance. In neuro-oncological surgery training, our review found a disproportionate focus on AR and VR simulations, with limited attention given to the potential of 3D-printed models and mixed reality.

The studies we reviewed often lacked objective metrics, relying primarily on subjective assessments. However, some research has made strides in incorporating objective performance measures. For example, AlZhrani et al. [3] evaluated neurosurgical performance during tumor resection using predefined proficiency benchmarks aimed at enhancing specific technical skills. The VR simulator NeuroTouch was utilized, categorizing performance metrics into Tier 1 (blood loss, tumor resection percentage, total simulated brain volume removed) and Tier 2 (instrument tip path length, force metrics, coordination indices).

The intrinsic complexity of neuro-oncological procedures poses significant challenges for traditional training, particularly for junior residents who may struggle to meet case load requirements necessary for skill development. Realistic simulators that integrate authentic manipulation experiences with onco-functional data could play a transformative role, enabling novices to build confidence, enhance their manual dexterity, and gain proficiency in interpreting complex anatomical structures (e.g., white matter and vascular anatomy) as well as using specialized instruments like Cavitron Ultrasonic Surgical Aspirator (CUSA) and neurostimulation tools. Emerging simulators are also targeting training in white matter anatomy and dissection, addressing the need for more effective tools in this complex area.

Despite these advancements, there is still no consensus on which specific skills are crucial for improving surgical outcomes due to limited evidence and a lack of standardized objective metrics. Among the reviewed studies, the majority demonstrated face validity, meaning that the simulation tools were perceived as realistic and useful by surgeons and trainees. Content validity was also commonly established, as most tools were designed to address critical neurosurgical competencies, including anatomical navigation, tumor resection planning, and intraoperative decision-making. However, only a limited number of studies provided construct validity by comparing expert and novice performance, which is crucial for determining whether simulation training accurately reflects different levels of surgical expertise. More importantly, very few studies assessed predictive validity, which evaluates whether simulation performance correlates with improved operative outcomes. Without robust predictive validity data, the ability of simulation tools to enhance surgical proficiency and patient safety remains largely theoretical.

Furthermore, evidence supporting the transferability of simulation training to the operating room was sparse. While some studies measured post-training improvements in technical skills and decision-making, only a handful explored the direct impact on intraoperative efficiency, complication rates, or surgical success. Future research should prioritize longitudinal studies that track the progression of trainees from simulation-based learning to real-world surgical performance, ensuring that these tools provide meaningful educational value beyond the simulated environment.

The ‘scarce ‘availability of neurosurgical simulation technology is an additional challenge too. Disparities in access across training centers raise concerns about the cost-effectiveness of these devices. Standardizing simulation training on a global scale is crucial, but questions remain about the optimal stage for implementation—whether during medical school or residency—and the appropriate number of training hours required. Additionally, ergonomic concerns, such as bulky VR headsets and cybersickness, must be addressed to enhance user experience and training efficiency. Recent advancements in cloud computing, 5G technology, and augmented reality (AR) have enabled the development of remote neurosurgical training systems. A study by Liu et al. [38] presents a highly immersive AR-based training framework that combines cloud computing and AR/VR technologies to offer real-time remote interaction. By integrating modules such as skin cutting, skull drilling, and peg transfer, this system has demonstrated significant improvements in surgical skill acquisition, particularly with AR outperforming VR in tactile realism and scene authenticity. Such remote training systems address key challenges, including the lack of access to in-person mentorship, and enable equitable training opportunities across geographically dispersed trainees. However, the adoption of these systems faces barriers such as the need for reliable internet infrastructure and validation of training outcomes in real-world clinical settings. Future work should explore how these innovations can be tailored and expanded to neuro-oncological surgery, ensuring their relevance to this subspecialty.

To summarize, the main challenges in the perioperative application of simulation technology in neuro-oncological surgery include technology-related disruptions to operative workflow, cost considerations, and the extent to which these tools can genuinely improve surgical outcomes. In terms of training, a lack of consensus on prioritizing which skills to develop, coupled with the absence of universally accepted objective metrics, continues to complicate their widespread implementation and routine use.

### Limitations

While our systematic review provides a comprehensive overview of the current state of the employment of simulation technology neuro-oncological surgery, several limitations are acknowledged. Firstly, the scope of the review was limited to studies published in English, Additionally, the heterogeneity of the study designs and outcome measures used in the included studies made it challenging to perform a meta-analysis or directly compare results across studies. Furthermore, many studies relied on subjective assessments of simulation efficacy rather than objective, quantitative measures, which may introduce bias and affect the reliability of the findings. An additional limitation of our study is the exclusion of studies focused on remote training. While such systems hold promise for enhancing neurosurgical training broadly, future reviews should incorporate these emerging technologies to provide a more comprehensive understanding of their potential.

## Conclusion

Our scoping review highlights the growing body of literature on simulation in neuro-oncological surgery. Simulation holds great potential for advancing neuro-oncological surgical training and perioperative planning, yet further research is required to widespread its clinical adoption and optimize its integration into neurosurgical education. Currently, while the number of studies is increasing, significant gaps remain in the depth of analysis and standardization of simulation methodologies.

## Electronic supplementary material

Below is the link to the electronic supplementary material.

Supplementary Material 1

## Data Availability

No datasets were generated or analysed during the current study.
