# Supplementary material for: Simulation tools in neuro-oncological surgery: a scoping review of perioperative and training applications
Source: J Neurooncol. 2025 Mar 19;173(1):21–35. doi: 10.1007/s11060-025-04972-8 (PMC12041155; doi:10.1007/s11060-025-04972-8)

**Supplementary Figure 1: Risk of bias in paper analyzing 3D models in pre-surgical planning**

**
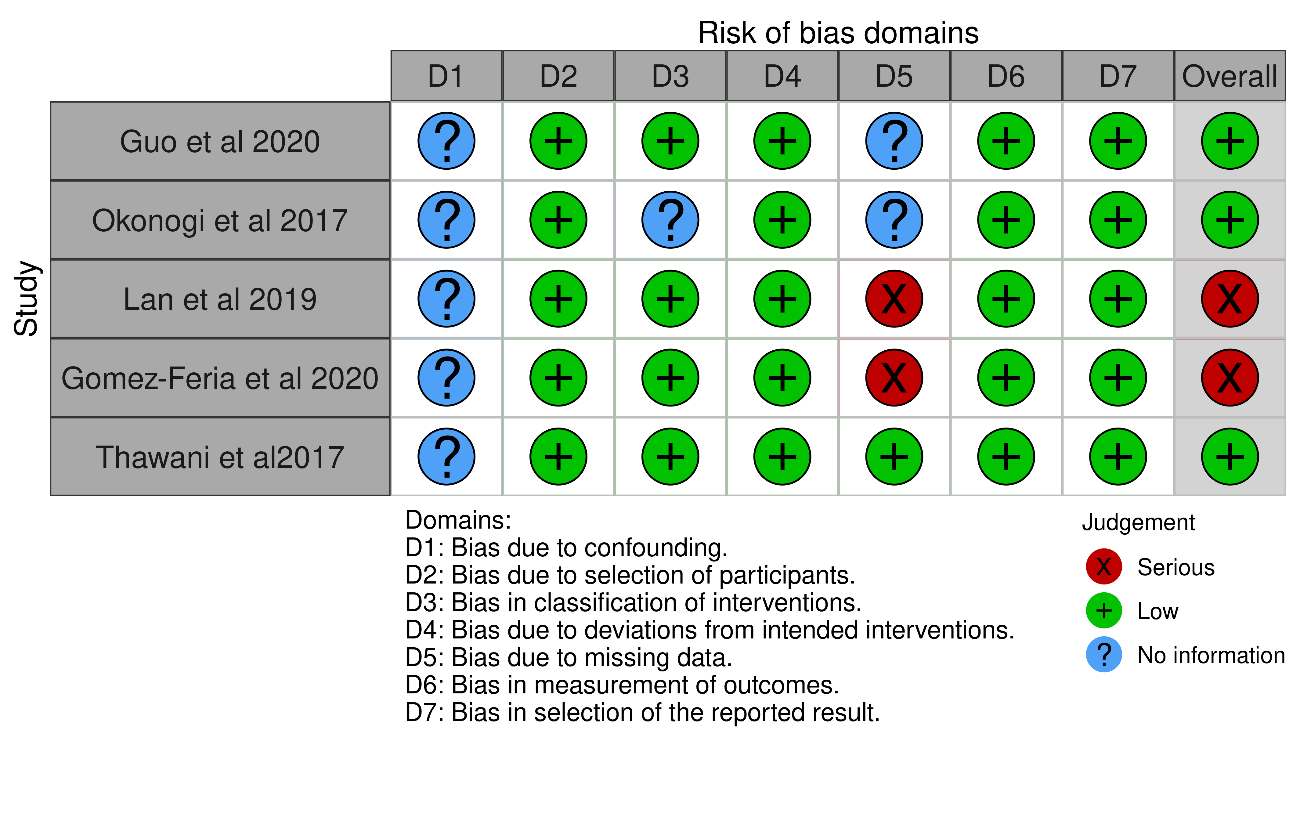
**

**Supplementary Figure 2: Risk of bias in paper analyzing use of AR in presurgical planning and intraoperative navigation**


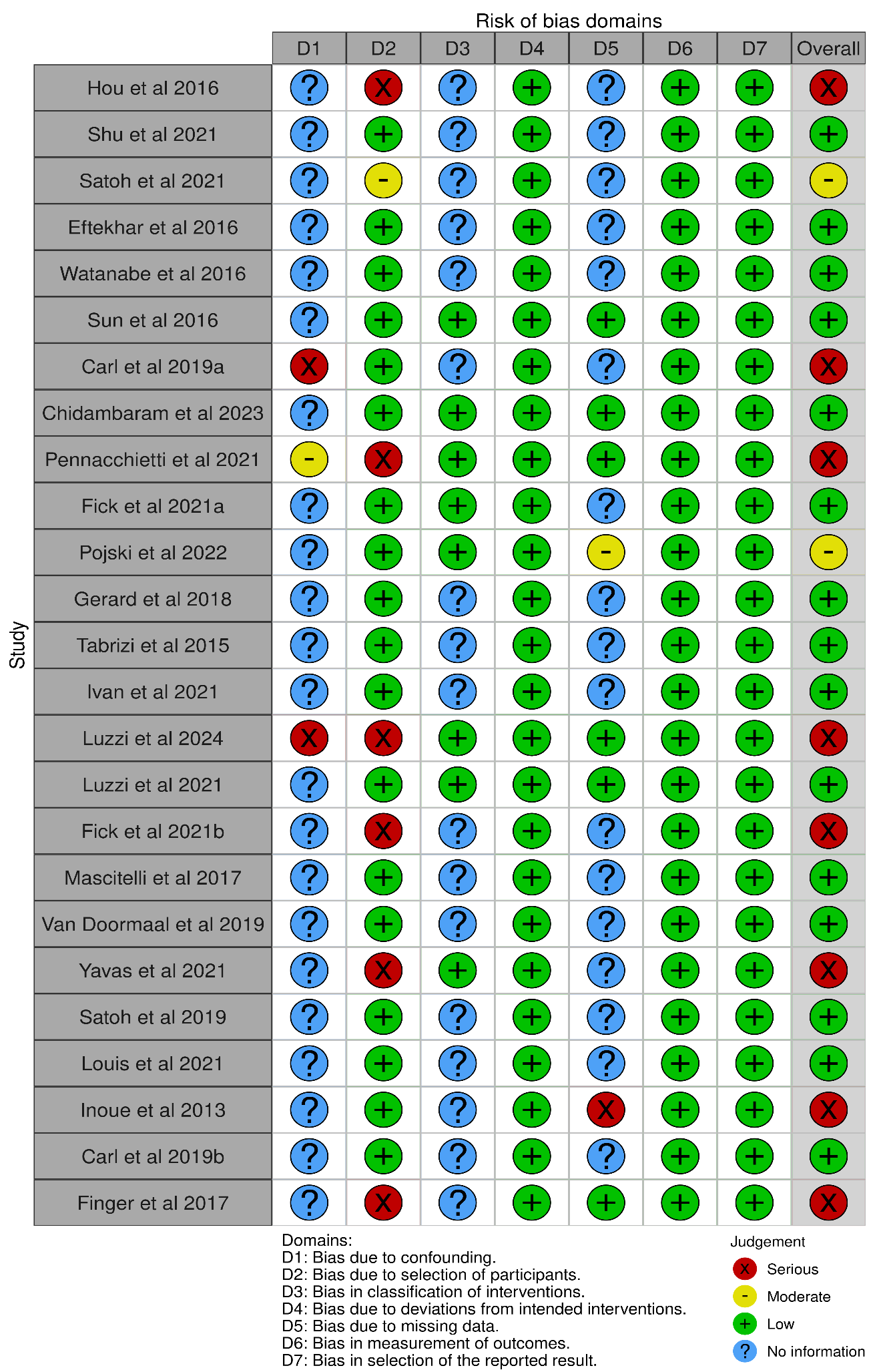

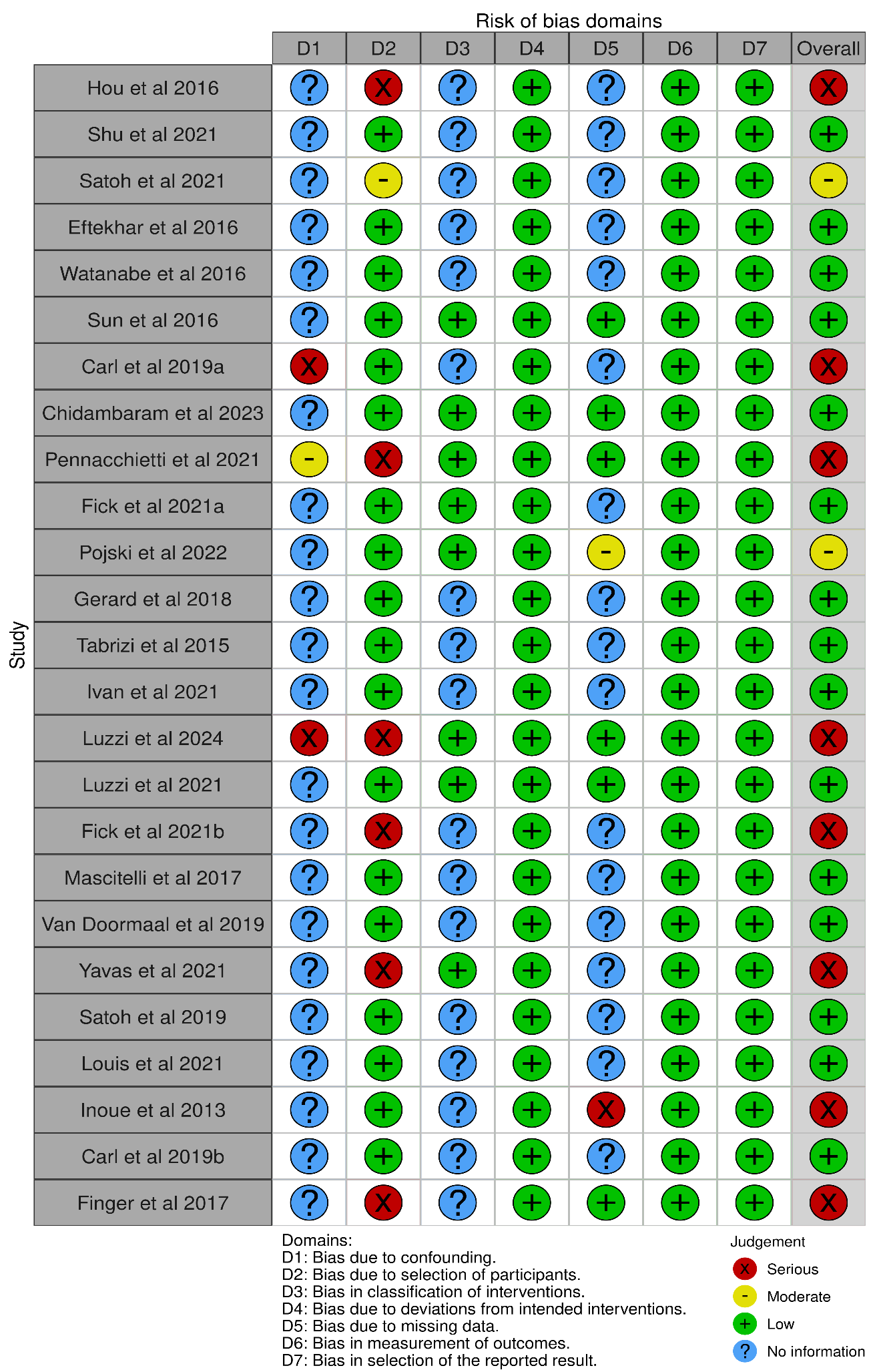


**Supplementary Figure 3: Risk of bias in paper analyzing use of MR in presurgical planning and intraoperative navigation**


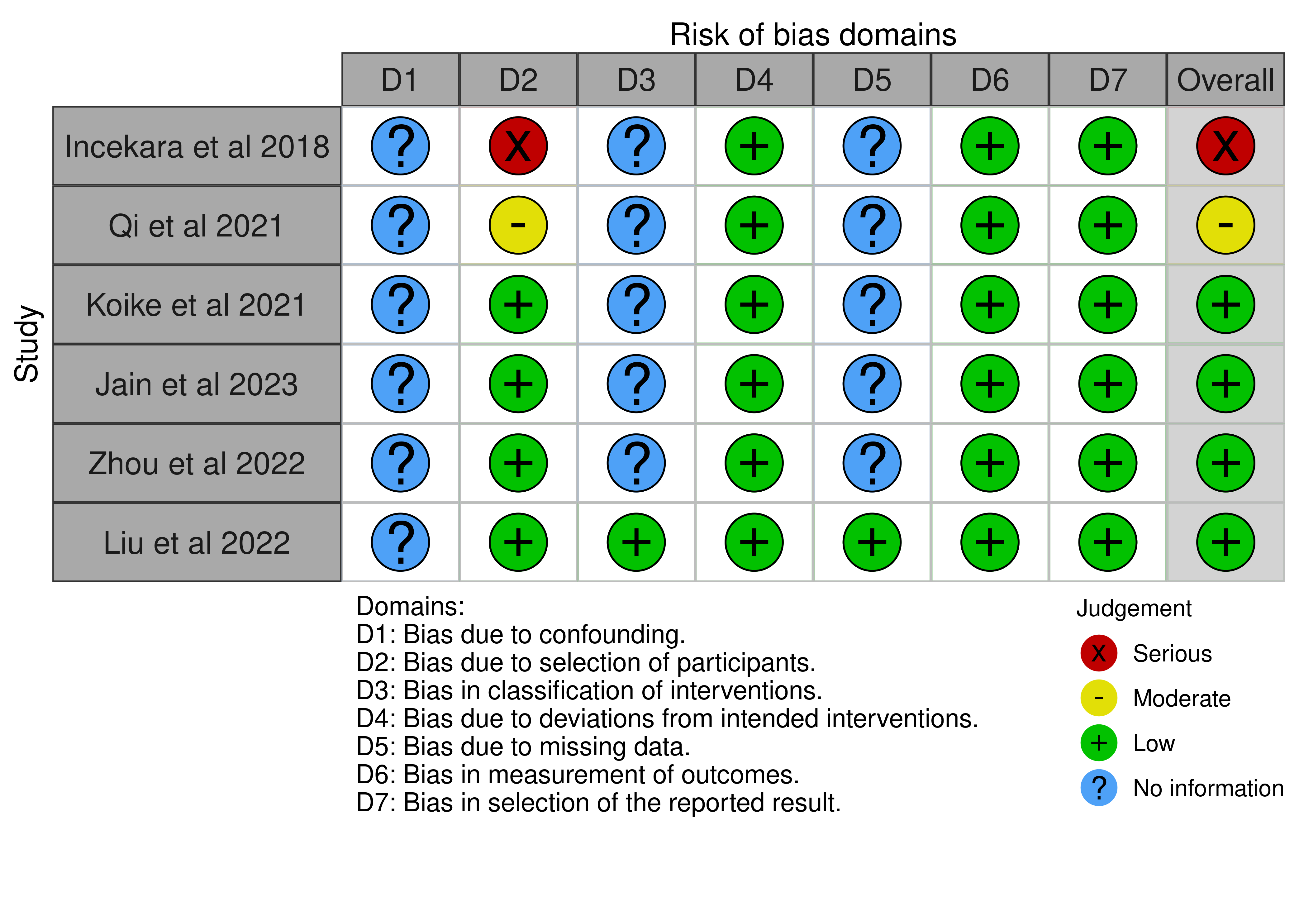


**Supplementary Figure 4: Risk of bias in paper analyzing use of VR presurgical planning and intraoperative navigation**


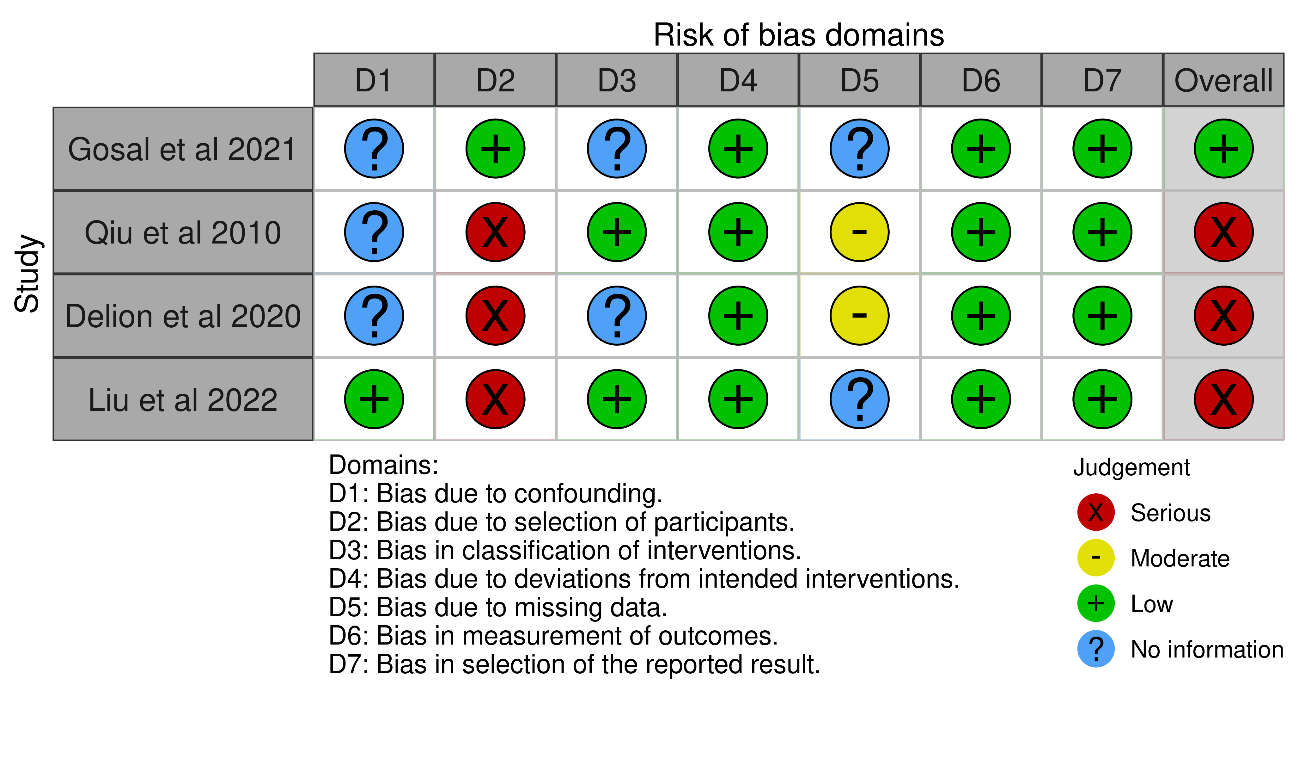

Supplement: Supplementary file 1 — Supplementary Material 1 [file 11060_2025_4972_MOESM1_ESM.docx]
